# Urinary glycoproteomic profiling of non-muscle invasive and muscle invasive bladder carcinoma patients reveals distinct N-glycosylation pattern of CD44, MGAM, and GINM1

**DOI:** 10.18632/oncotarget.27696

**Published:** 2020-08-25

**Authors:** Gajanan Sathe, Irene A. George, Barnali Deb, Ankit P. Jain, Krishna Patel, Brusabhanu Nayak, Subhradip Karmakar, Amlesh Seth, Akhilesh Pandey, Prashant Kumar

**Affiliations:** ^1^Institute of Bioinformatics, International Technology Park, Bangalore 560066, India; ^2^Manipal Academy of Higher Education (MAHE), Manipal 576104, India; ^3^School of Biotechnology, Amrita Vishwa Vidyapeetham, Kollam 690525, India; ^4^Department of Urology, All India Institute of Medical Sciences, New Delhi 110070, India; ^5^Department of Biochemistry, All India Institute of Medical Sciences, New Delhi 110070, India; ^6^Center for Molecular Medicine, National Institute of Mental Health and Neurosciences (NIMHANS), Bangalore 560029, India; ^7^Department of Laboratory Medicine and Pathology, Center for Individualized Medicine, Mayo Clinic, Rochester, MN 55905, USA; ^*^These authors contributed equally to this work and share the first authorship; ^#^These authors contributed equally to this work and share the second authorship

**Keywords:** urothelial carcinoma, N-linked glycoproteomics, urine proteomics, NMIBC, MIBC

## Abstract

Clinical management of bladder carcinomas (BC) remains a major challenge and demands comprehensive multi-omics analysis for better stratification of the disease. Identification of patients on risk requires identification of signatures predicting prognosis risk of the patients. Understanding the molecular alterations associated with the disease onset and progression could improve the routinely used diagnostic and therapy procedures. In this study, we investigated the aberrant changes in N-glycosylation pattern of proteins associated with tumorigenesis as well as disease progression in bladder cancer. We integrated and compared global N-glycoproteomic and proteomic profile of urine samples from bladder cancer patients at different clinicopathological stages (non-muscle invasive and muscle-invasive patients [*n* = 5 and 4 in each cohort]) with healthy subjects (*n* = 5) using SPEG method. We identified 635 N-glycopeptides corresponding to 381 proteins and 543 N-glycopeptides corresponding to 326 proteins in NMIBC and MIBC patients respectively. Moreover, we identified altered glycosylation in 41 NMIBC and 21 MIBC proteins without any significant change in protein abundance levels. In concordance with the previously published bladder cancer cell line N-glycoproteomic data, we also observed dysregulated glycosylation in ECM related proteins. Further, we identified distinct N-glycosylation pattern of CD44, MGAM, and GINM1 between NMIBC and MIBC patients, which may be associated with disease progression in bladder cancer. These aberrant protein glycosylation events would provide a novel approach for bladder carcinoma diagnosis and further define novel mechanisms of tumor initiation and progression.

## INTRODUCTION

Bladder carcinoma is one of the most common urothelial cancers worldwide with high morbidity and mortality of 2,000,000 (GLOBOCAN, 2018). Patients are predominantly diagnosed with non-muscle invasive bladder cancer carcinoma (NMIBC) is the prevalent type (70–75%) and is defined as a superficial neoplasia. These tumors are restricted to the mucosal or sub mucosal layer with high risk of recurrence [1]. About, 10–20% of NMIBC will progress into muscle invasive bladder carcinoma (MIBC) which has a high risk of metastasis and is characterized by poor prognosis [2]. Accurate staging, grading, risk stratification is essential for disease management and better clinical outcome of the patients [3]. Presently the major diagnosis methods of bladder carcinoma are imaging tests (such as ultrasound, CT, MRI), cystoscopy, and urine cytology. However, they lack either precision or sensitivity mostly in NMIBCs. Hence, high recurrence rate and high costs in clinics are the major challenges in the management of this cancer. Identifying the precise, non-invasive, economical, and convenient methods as well as a highly robust biomarker for bladder carcinoma diagnosis is the booming need of this era. The best choice a novel non-invasive, affordable method for diagnosis, as well as for monitoring disease progression after treatment as well as for predicting prognosis would be through urine-based detection methods [4].

Glycosylation of proteins has an impact on many biological functions such as protein folding, trafficking, endocytosis, cell adhesion, receptor activation, immune functions [5]. Altered glycosylation has been aligned with carcinogenesis, progression of the disease, tumor associated invasion, sensitivity to treatment [6–9]. Cancer specific changes in glycosylation can serve as diagnostic as well as therapeutic targets, for instance aberrant N-glycosylation of serum Immunoglobulins (Igs) urothelial carcinoma suggests glycosylation pattern as diagnostic biomarker [10, 11]. Dysregulation of protein glycosylation has been associated with cancer hallmarks in bladder cancer [12, 13]. Dysregulated glycosylation of extracellular matrix proteins has been reported in aggressive non-type subtype bladder cancer cells compared to basal/ luminal type [14].

In this study, we have compared the urinary N-glycoproteomic and proteomic profile of NMIBC and MIBC along with control groups. We employed tandem mass tag (TMT) labelling method for quantitation along with the solid phase extraction of N-linked glycopeptide (SPEG) for the enrichment. Combining these cutting-edge sample preparation techniques with high resolution mass spectrometry enable us to identify urinary proteomic and N-glycosylation alterations in the NMIBC and MIBC. To our knowledge, this is the first report on the combining proteomic and N-glycoproteomic analysis from urine from muscle invasive and non-invasive bladder cancer patients and control samples.

## RESULTS

### Global proteomic and N-glycoproteomic profiling of bladder carcinoma patients

**Figure 1:**
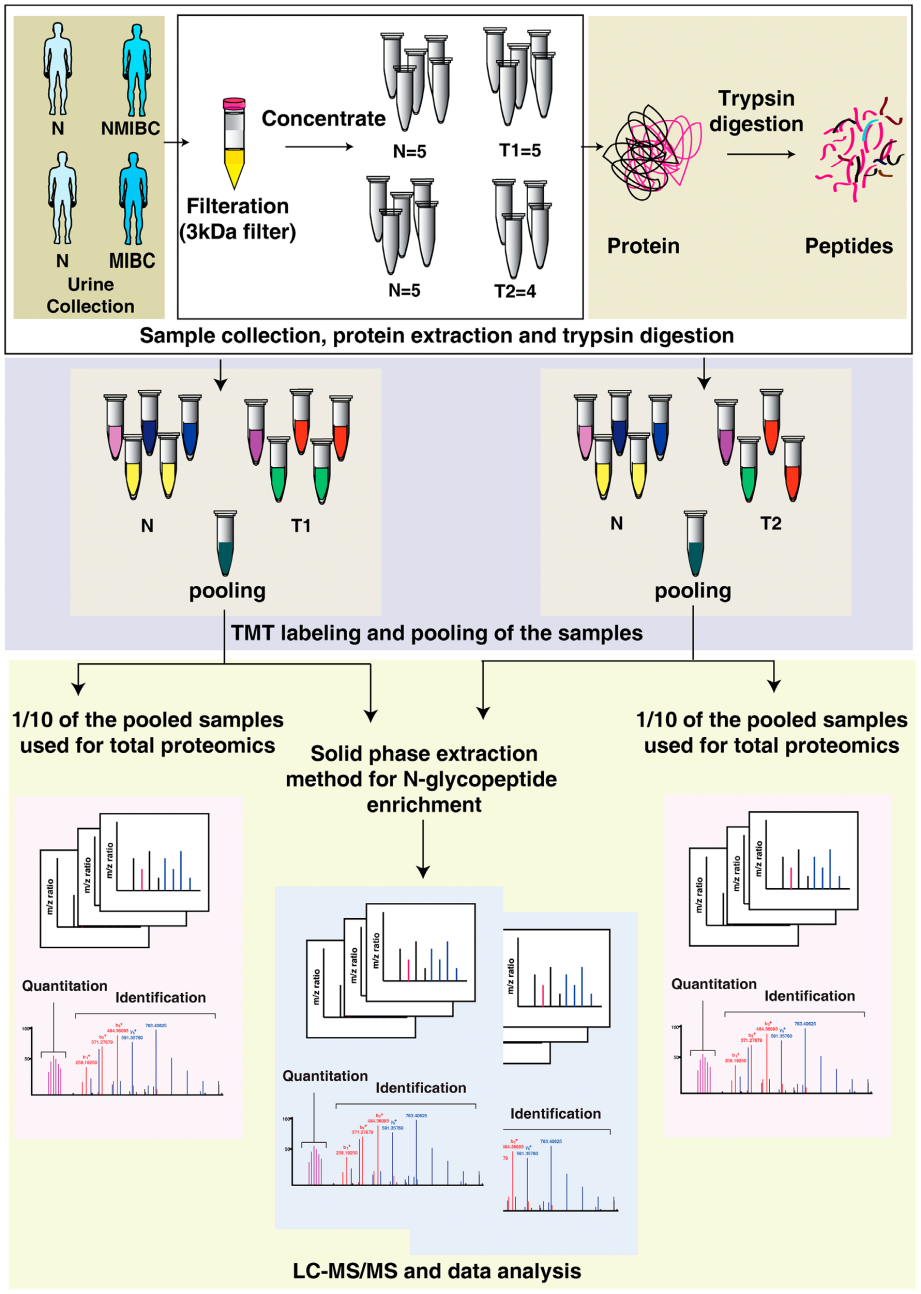
Integrated proteomics and N-glycoproteome analysis workflow applied to urine samples from bladder cancer patients and healthy subjects. Following filtration with 3kDA filters, urinary proteins were concentrated and digested with trypsin. Each sample was labeled with 10-plex Tandem Mass Tags (TMT) labeling kit and pooled. One-tenth of the pooled sample was used for global proteomics experiment and remaining was enriched for N-glycoproteomic analysis. Samples were analyzed in Q Exactive HF-X Hybrid Quadrupole-Orbitrap mass spectrometer and the files were searched against Mascot and Sequest HT search engines. Data analysis was carried out using Perseus. exe 1.6.5.0.

For the understanding of the alteration in the proteome and N glycosylation associated with bladder carcinoma, we performed LC-MS/MS-based quantitative N-glycoproteomics and proteomics analysis of urine samples collected from healthy subjects (*n* = 5), non-muscle invasive (*n* = 5) and muscle invasive (*n* = 4) bladder carcinoma patients. The work flow of the experiment is schematically illustrated in Figure 1. We identified a total of 590 proteins and 763 N-glycopeptides (Supplementary Figure 1A and 1B).

### Global quantitative urinary N-glycoproteomic and proteomic analysis of non-muscle invasive bladder cancer patients

**Figure 2:**
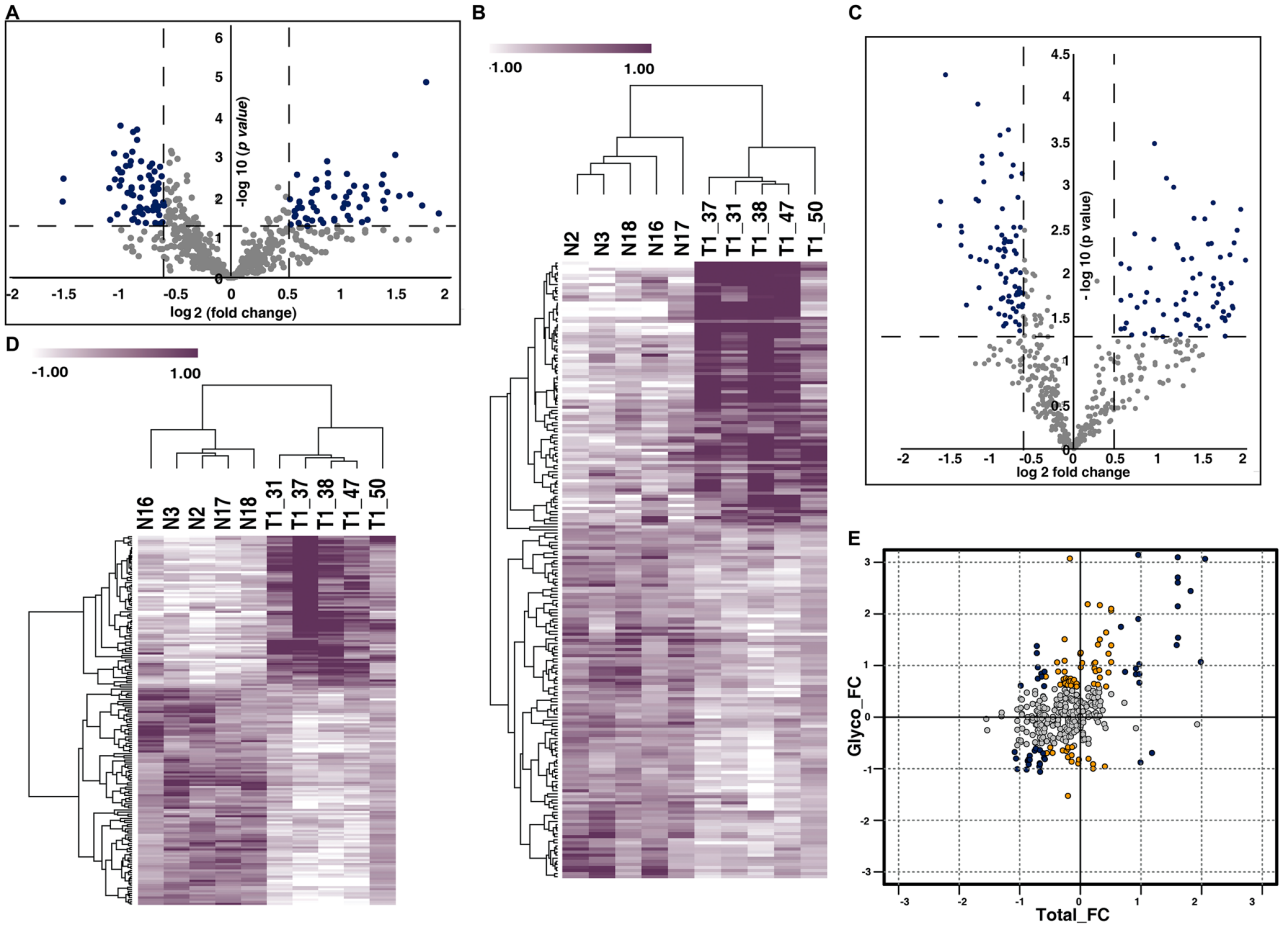
Urinary N-glycoproteome and proteome of NMIBC. (**A**) Volcano plot of N-glycoproteome of NMIBC and normal subjects. Fold change of the N-glycosylation abundances (log2) are plotted against the *t*-test *p*-values (-log10). Significance levels are marked by dashed lines. (**B**) Unsupervised clustering of dysregulated N-glycopeptides identified in NMIBC and healthy subjects. *t*-test between the two cohorts revealed dysregulation of N-glycosylation in 201 glycopeptides. Abundance values of differentially glycosylated peptides are represented in heatmap. (**C**) Volcano plot represents the proteomic profile of NMIBC and healthy subjects. Fold change of proteins (log2) expressed in NMIBC patients were calculated with respect to the healthy subjects and plotted against the *t*-test *p*-value (-log10). Dashed lines represent the significance levels. (**D**) Heat map represents the abundances of differentially expressed proteins between the NMIBC and healthy subjects. (**E**) Global presentation of proteomic and respective glycosylation occupancy. Yellow datapoints represent differential glycosylation occupancy with fold change ≥ 1.5 and blue datapoints with fold change ≥ 2 on proteins that are unchanged in proteomic data. Grey datapoints represent proteins that are unchanged in both datasets.

We compared the N-glycoproteomic and proteomic profile of urine samples from NMIBC patients and the healthy subjects. N-glycopeptide enrichment of the pooled labeled samples were carried out using solid phase extraction of glycopeptides (SPEG). This leads to the identification of 635 N-linked glycopeptides corresponding to 381 proteins. We quantified 625 glycopeptides corresponding to 378 glycoproteins across all the samples (Supplementary Table 2) (Figure 2A). From the quantified glycopeptides we observed differential N-glycosylation of 201 N-linked glycopeptides corresponding to 164 proteins (*p*-value < 0.05). Unsupervised clustering revealed distinct N-glycosylation pattern between the cohorts (Figure 2B).

From our global proteomic data, we identified 543 proteins of which 485 proteins are quantified across all the samples (Supplementary Table 3). The quantified proteins are represented in volcano plot (Figure 2C). Statistical analysis (*t*-test) revealed 177 proteins to be significantly altered (*p*-value < 0.05) between the cohorts (Figure 2D).

To identify if the changes were because of alteration in protein abundance or in glycosylation site occupancy, we compared the glycoprotein changes identified from our glycoproteomic analysis to those identified from our global proteomic analysis. We compared the N-glycosylation fold change against the total protein fold change. We identified 41 differentially glycosylated proteins without any changes in protein abundance (Figure 2E).

### Quantitative urinary proteomic and glycoproteomic analysis of muscle invasive bladder carcinoma patients

**Figure 3:**
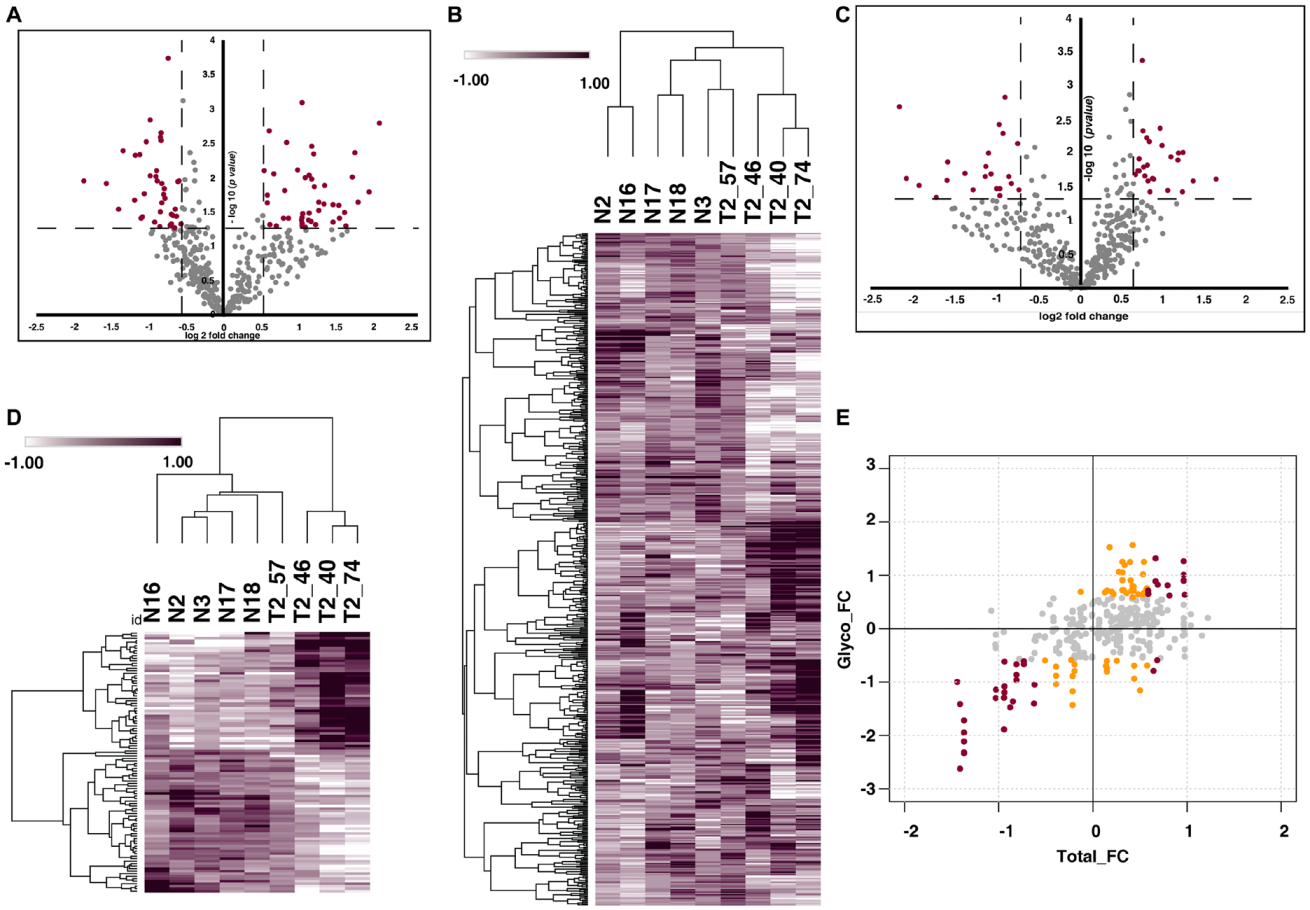
Urinary N-glycoproteome and proteome of MIBC. (**A**) Volcano plot shows the N-glycoproteome profile of MIBC and healthy subjects. Fold change of the N-glycosylation abundances (log2) are plotted against the *t*-test *p*-values (-log10). Significance levels are indicated by dashed lines. (**B**) Heat map representing the significantly glycosylated peptides in MIBC group in comparison with the healthy subjects (*p*-value < 0.05). Abundance values of the differentially glycosylated peptides are represented in the heat map. (**C**) Volcano plot represents comparative proteomic profile of MIBC and healthy subjects. Fold change of the protein abundances versus *t*-test *p*-values (-log10). Significance levels are indicated by dashed lines. (**D**) Heat map of differentially expressed proteins (log2 abundance value) of MIBC compared with the healthy subjects. (**E**) Global presentation of proteomic and respective glycosylation occupancy. Yellow datapoints represent differential glycosylation occupancy with fold change ≥ 1.5 and red datapoints with fold change ≥ 2 on proteins that are unchanged in proteomic data. Grey datapoints represent proteins that are unchanged in both datasets.

We further compared N-glycoproteomic and proteomic profiling of MIBC patients with healthy individuals. Glycoproteome profiling of MIBC patient urine samples resulted in identification of 543 N-glycopeptides corresponding to 326 proteins and quantified 537 glycopeptides (Supplementary Table 4), which mapped to 322 proteins (Figure 3A). From the quantified 537 peptides we identified of 82 glycopeptides corresponding to 65 were differentially glycosylated in MIBC compared to healthy group (*p*-value < 0.05) (Figure 3B).

From our global proteomic data, we identified 486 proteins and 427 (Supplementary Table 5) proteins are quantified across all the samples (Figure 3C). Further, 72 quantified proteins (*p*-value < 0.05) were differentially expressed (Figure 3D). The integrative analysis of the proteomic and glycoproteomic data revealed differential glycosylation of 21 proteins without any change at total protein expression level (Figure 3E).

### Comparison of N-glycoproteomic patient data with previously published cell line data

**Figure 4:**
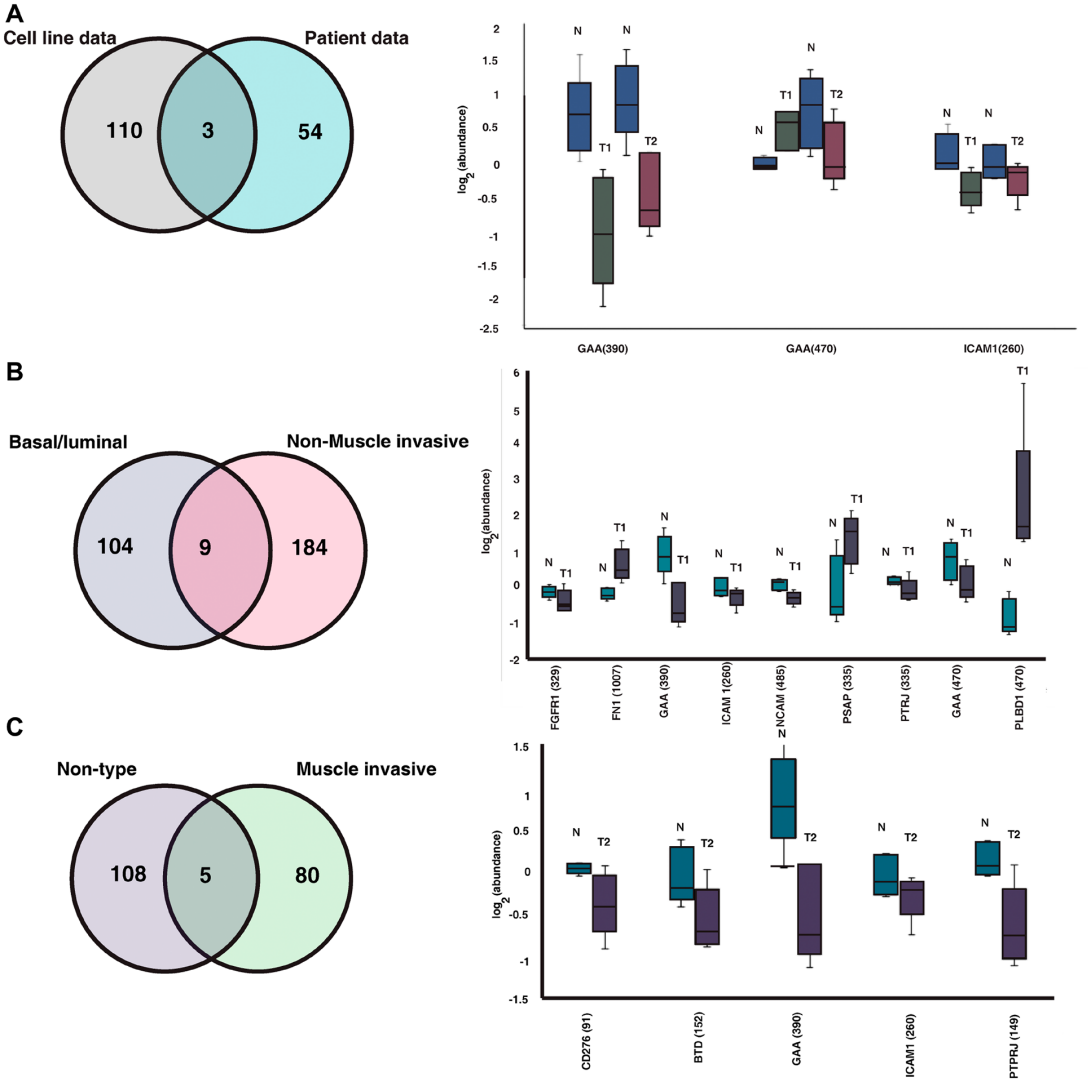
Comparison between cell line and our clinical data. (**A**) Comparison between the previously published cell line data and the clinical data identified site-specific glycosylation of 3 proteins common between the data. Box plot represents the regulation of the proteins in healthy subjects, NMIBC (T1) and MIBC (T2) patients. (**B**) Comparing the N-glycoproteome of basal/luminal bladder cancer cell lines and the NMIBC patients identified site specific glycosylation of 9 proteins. Regulation of the glycosylation between the NMIBC and healthy subjects are shown in box plot. (**C**) Comparing the N-glycoproteome of non-type bladder cancer cell lines and the NMIBC patients identified site specific glycosylation of 5 proteins. Regulation of the glycosylation between the NMIBC and healthy subjects are shown in box plot.

In our previous study by Deb *et al*., we reported distinct glycosylation profile between the basal/luminal and non-type bladder cancer cell lines [14]. To assess the concordance between the urinary N-glycoproteome data along with previously published cell line data, we compared the cell line data with the commonly expressed glycosylation site between NMIBC and MIBC. We identified site-specific glycosylation in 2 sites of GAA (390, 470) and 1 site of ICAM1 (260) (Figure 4A). We further compared the glycosylation profile of less aggressive basal/ luminal with NMIBC patients and aggressive non-type cell lines with MIBC patients. We found that 9 site specific glycosylation of FGFR1 (329), FN1 (1007), GAA (390), ICAM1 (260), NCAM1 (485), PSAP (335), PTPRJ (335), GAA (470) and PLBD1 (470) are common for basal/luminal cell line vs NMIBC patient data (Figure 4B). Similarly, we also compared the non-type cell line data with MIBC patient data and identified 5 site-specific glycosylation across the groups. N-glycosylation of the all the 5 proteins namely, CD276 (91), BTD (152), GAA (390), ICAM1 (260) and PTPRJ (149) were downregulated in MIBC compared with the control groups (Figure 4C).

### Comparison between the N-glycoproteome and proteome of NMIBC and MIBC patients

**Figure 5:**
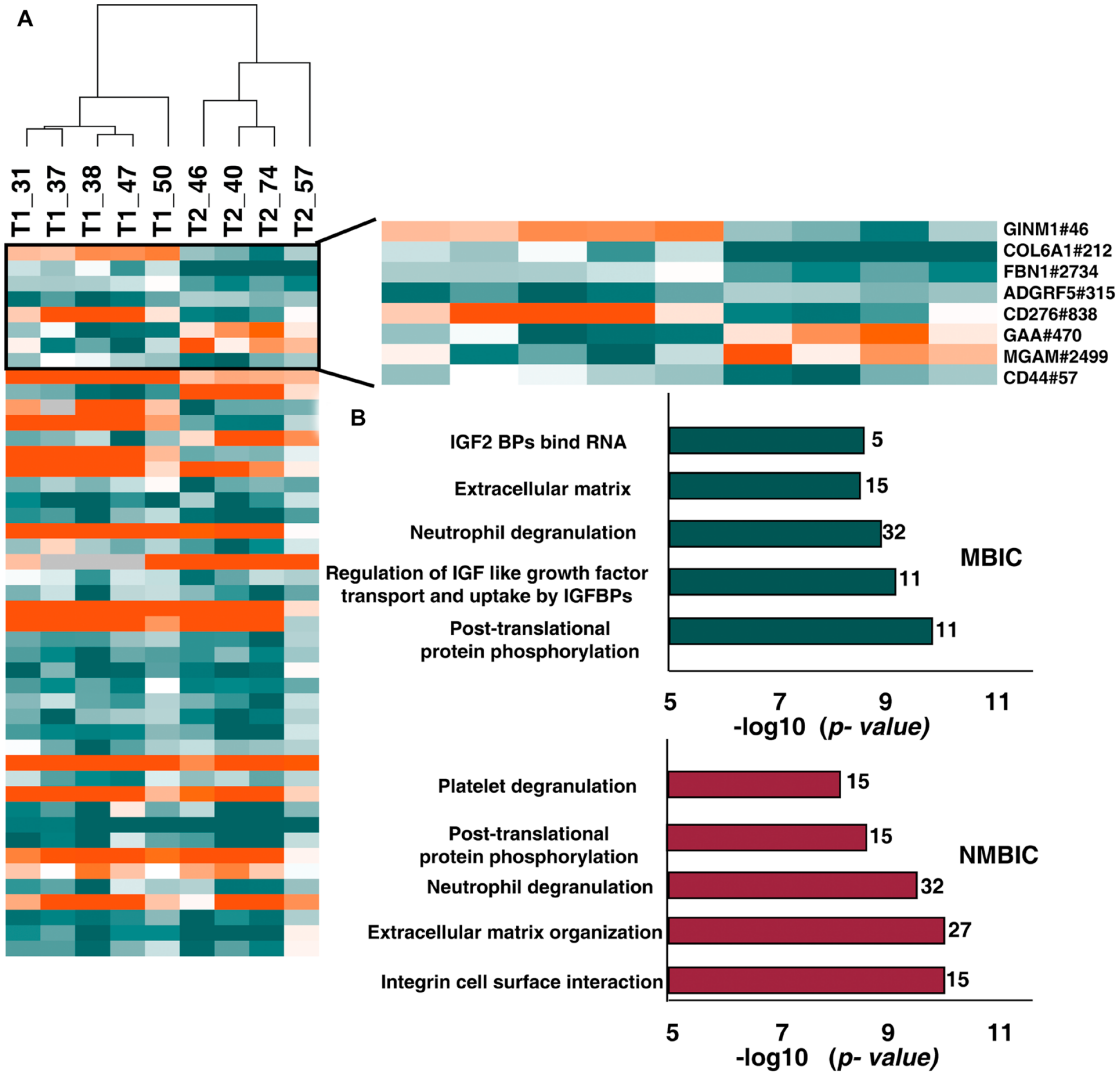
Comparison between N-glycoproteome and proteome of NMIBC and MIBC patients. (**A**) Heatmap representing the differentially glycosylated peptides in NMIBC and MIBC patients. Highlighted are the proteins with significant glycosylation (> 2-fold) without any change in protein expression level. (**B**) Top 5 enriched pathways in NMIBC and MIBC patients. Reactome analysis identified integrin cell surface interaction as most enriched in NMIBC and post-translational modification as most enriched pathway in MIBC.

Comparing the N-glycoproteome and global proteomic expression could reveal the stage specific glycoproteomic and protein expression pattern in bladder carcinoma patients. We compared the differentially glycosylated peptides between NMIBC and MIBC and identified glycosylation of 46 N-glycopeptides corresponding to 41 proteins (Figure 5A, Supplementary Table 6). Statistical analysis of the commonly glycosylated proteins revealed significant alteration of glycosylation in 8 proteins between NMIBC and MIBC. Of these proteins such as COL6A1(212), FBN1(2734), GAA (470) show significant glycosylation without any change in protein expression both in NMIBC and MIBC patients. Glycosylation of GINM1(46) is downregulated in NMIBC without any change in protein expression level. We observed altered glycosylation of CD44 (57) and MGAM (2499) in MIBC patients without any significant change in protein expression.

### Reactome pathway analysis revealed dysregulation of oncogenic pathways in bladder carcinoma

We further carried out Reactome pathway analysis with the 164 differentially glycosylated proteins in NMIBC and 65 differentially glycosylated proteins of MIBC. Top 5 enriched pathways are listed in Figure 5B. Pathways such as “extracellular matrix organization” (*p*-value = 9.57E-11, 4.6E-9; FDR = 4.19E-08, 5.84E-7), “post-translational protein phosphorylation” (*p*-value = 2.22E-09, 1.99 E-10; FDR = 4.87E-07, 1.01 E-7) and neutrophil degranulation (*p*-value = 2.90E-10, 2.36E-09; FDR = 8.47E-08, 3.99E-07) were dysregulated in NMIBC as well as MIBC patients (Supplementary Table 7).

## DISCUSSION

Bladder carcinoma management is contingent on stratification of patients based on their clinicopathological subtype, probability of recurrence and progression of the disease. Molecular complexity is often a major contributor for creating a hindrance in clinical diagnosis as well as treatment resolutions. PTMs, add the complexity of a protein, however, they also potentially offer novel diagnostic and targeting opportunities across tumor sub-populations. Profiling these PTMs such as glycosylation from cancer patients’ samples would eventually lead to expanding the knowledge repertoire from better mechanistic studies. Hence, in this study, we carried out comparative N-glycoproteomic and proteomic profiling of urine samples from NMIBC and MIBC patients, as it is the most widely used non-invasive specimen for BC diagnosis and monitoring. Here, we utilized TMT-based proteomic approach to demonstrate the dysregulated glycosylation and differentially expressed proteins in BC.

For a comprehensive glycosylation profile of BC and dissecting the unique patterns in NMIBC and MIBCs, combining the global protein expression data was inevitable. Thus, we integrated the glycoproteomic and proteomic data of NMIBC patients and among the 41 proteins underwent glycosylation without any significant changes in protein expression levels. Metalloproteinase-8 (MMP8), cluster of differentiation 59 (CD59), apolipoprotein H (APOH), fibrinogen beta chain precursor (FGB), Fibrillin-1 (FBN1), and S100A1, a few to mention. High mRNA level of MMP8 has been reported in bladder cancer and is positively correlated with tumor grade [15]. In this study, we observed MMP8 is heavily glycosylated in NMIBC patients compared to the healthy subjects. High expression of FBN1 has been reported in bladder cancer patients and associated with worst overall survival [16]. FBN1 is also known to induce metastasis in ovarian cancer [17]. Recently, the role of complement system in cancer progression is widely studied. The complement factor C3, which is known to be associated with tumor progression is highly glycosylated in our study. It has been hypothesized that cancer cells escape from complement system by activating complement inhibitors, such as CD59, CD46, and CD55. The membrane complement inhibitor CD59 expression has been reported to be upregulated in many cancers including bladder and is correlated with cell proliferation, apoptosis and immune evasion [18–21]. The altered glycosylation in complement system proteins and their inhibitors might be aiding the tumor cells to evade the immune system and leading to tumorigenicity.

In addition, comparison between the glycoproteomic and proteomic data of MIBC patients revealed the dysregulation of glycosylation in 21 proteins without any alterations in protein expression. Some of these proteins include, vitronectin (VN), fibrinogen beta chain protein (FGB), myeloperoxidase (MPO), serpin family C member 1 (SERPINC1), cadherin13 (CDH13). VN that functions as an adhesive molecule between the cells and ECM has been reported in cancers for cell growth, angiogenesis and metastasis [22]. A recent study has demonstrated VN is a potent promigratory factor and its activity is inhibited by fibrinogen [23]. In our data, we observed altered glycosylation of VN and FGB without any significant change in protein expression level. Przybyło *et al*., postulated altered glycosylation in cadherins interrupt the effective interactions between the cells in bladder cancer [24]. Downregulation of CDH13 expression promotes invasiveness of bladder transitional cell carcinoma [25].

In this study, we conducted a broad analysis of the differentially expressed proteins as well. Interestingly, altered expression levels of ECM and immune system related proteins were observed in NMIBC data. MMP-9 was elevated in the urine samples of BC patients and has been associated with tumor growth, invasion and metastasis [26, 27]. High expression of complement proteins has been associated with tumor growth, evasion from immune surveillance, metastasis and EMT in various cancers [28]. Complement proteins such as C3, C4A, CFB were highly expressed in NMIBC patient samples. Altered N-glycosylation and expression of these proteins may promote carcinogenesis by regulating tumor initiating events such as proliferation and immune evasion in BC. We also observed high expression of S100 family proteins such as S100A8, S100A9, S100P and S100A12 in MIBC patients. Altered expression of S100 proteins has been correlated with progression of various cancers [29, 30].

In our previous study by Deb *et al.,* we carried out N-glycoproteomic profiling of basal/luminal as well as aggressive non-type bladder carcinoma cell lines. Glycoproteomic profiling of bladder cancer cell lines identified altered glycosylation of extracellular matrix associated proteins in non-type cells compared with the basal/luminal cells [14]. We compared our patient data with the cell line data to check the concordance of cell line and clinical data sets. Interestingly, we identified site-specific glycosylation of 9 proteins between NMIBC and basal/luminal cell lines and 5 between the MIBC patients and non-type cell lines. Higher glycosylation of FN1 was observed in NMIBC patient cohort and its glycosylation has been reported to be involved in positive regulation of cell adhesion and directing cell migration [31]. ICAM1, which is identified in cell lines as well as patients’ urine data, is known to promote tumorigenesis and increase the metastatic potential of cancer cells [32].

Further, to identify the pattern of N-glycosylation and protein expression associated with disease progression we compared the N-glycoproteome as well as proteome data of NMIBC and MIBC patients. However, we could not identify significant number of proteins with a unique glycosylation trend across the NMIBCs or MIBCs. This is also quite intuitive that MIBCs and NMIBCs would have a similar molecular profile as they are progression stages and though the clinical manifestation is distinct it may not reflect similarity in molecular regulation. We found significant glycosylation in proteins such as fibrillin-1 (FBN1), collagen type VI alpha 1 chain (COL6A1) and glucosidase alpha acid (GAA) both in NMIBC and MIBC patients. Survival analysis of bladder cancer patients revealed elevated expression COL6A1 and FBN1 is associated with a worse overall survival and progression of disease [16]. We observed unique glycosylation of glycoprotein integral membrane 1 (GINM1) in NMIBC patients, however the role of this protein in cancer development or progression is not yet reported. We also observed unique glycosylation of maltase-glucoamylase alpha-glucosidase (MGAM) and cluster of differentiation 44 (CD44) in MIBC patients. CD44 is overexpressed in several cancers and is a molecular marker for stem cells [33, 34]. High expression of CD44 has been associated with higher clinical stage and aggressiveness in bladder cancer [35]. CD44 can bind to its ligand hyaluronic acid (HA) and is known to activate cell proliferation, invasion, migration [33]. It has been shown that changes in glycosylation of CD44 can affect its interaction with HA and glycosylation may also be a regulatory mechanism for CD44 function [36]. Also, CD44 is involved in activating various oncogenic events in cancer tissues through activation of signaling pathways such as PI3K/ AKT and RhoGTPase. Activation of these pathways promotes angiogenesis, survival, invasion and promotes tumor progression [37]. The unique glycosylation of CD44 in MIBC patients may account for the tumorigenic and tumor progression processes, however further validations has to be carried out.

The pathway analysis of NMIBC patients revealed dysregulation of pathways such as integrin cell surface interaction, extracellular matrix organization, neutrophil degranulation, post translational protein phosphorylation and platelet degranulation as top five. In MIBC patients we observed dysregulation of pathways such as post translational protein phosphorylation, regulation of IGF like growth factor transport and uptake by IGFBPs, neutrophil degranulation, extracellular matrix organization and IGF2 bind RNA. Among these extracellular matrix organization pathway and neutrophil degranulation pathways are found to be dysregulated in both stages. The role of ECM proteins in development of invasion, progression and metastasis of bladder cancer is widely studied [38].

We observed a clear difference between the glycosylation profile and protein expression among the control and tumor groups. We hypothesized that differential glycosylation of proteins between the NMIBC results in tumorigenic processes whereas the differential glycosylation in MIBC may be associated with tumor progression. However, most of the differentially glycosylated proteins are commonly dysregulated in NMIBC and MIBC patients suggesting an overlap of molecular events of the disease.

## MATERIALS AND METHODS

### Patient sample collection

Urine samples were collected from patients confirmed with bladder carcinoma enrolled in All India Institute of Medical Science (AIIMS), Delhi. The urine for the control group is collected from healthy volunteers with consent. The study was conducted in accordance with the ethical requirements and the project was approved by the Ethics Committee (Project identification code: IEC-372/07.07.2017). Voided urine samples and associated clinical information is summarized in Supplementary Table 1. For the initial screening, voided urine was collected from healthy subjects and bladder carcinoma patients in 50 ml centrifuge tubes. The voided urine was clean catch, midstream urine, and the samples did not contain squamous cells or microbes. The samples collected from the healthy individuals (the control group), were confirmed with no previous history of urothelial cell carcinoma, gross hematuria, active urinary tract infection or urolithiasis. After collection, 15 ml urine from each sample was centrifuged at 7000 g for 15 minutes at 4°C to remove the cell debris. Supernatant was collected, concentrated to 500 μl in 3 kDa filters. The concentrated urine was stored at –80°C until use.

### Protein digestion and TMT peptide labeling

Protein amount was estimated using BCA (Bicinchoninic Acid) protein assay. From all samples 100 μg of protein from each sample were reduced, alkylated and precipitated with 10 mM (dithiothreitol) DTT, 20 mM iodoacetamide (IAA) and ice-cold acetone. The precipitated samples were then resuspended in 50 mM triethyl ammonium bicarbonate (TEABC) and digested with trypsin (1:20, Promega) 12–16 h at 37°C. Resulting peptides were cleaned using Sep-Pak C18 material (Waters, Catalog # WAT023501). Peptides from corresponding samples were labeled with 10 plex TMT labels according to manufacturer’s protocol.

### N-Glycan enrichment

Glycopeptides were enriched directly by employing solid-phase extraction of N-glycopeptides method as described previously [39]. Glycopeptides from the tryptic peptide digest were enriched using solid-phase extraction of glycosite-containing peptides (SPEG). Briefly, 90% of the TMT-labeled tryptic peptides (1 mg) were dissolved in 5% ACN in 0.1% TFA followed by adding 1/10 of the final volume of 100 mM sodium periodate to the samples. These were incubated in the dark at room temperature for 1 hour with gentle shaking. After this step, peptides were cleaned using C18 clean-up. For the experiment, 200 μL of the 50% hydrazide resin (Bio-Rad Laboratories, Hercules, CA, USA) were used. Hydrazide resin beads were further washed three times with 1 ml deionized water. Following this, peptides along with 1% Aniline were added to the beads (pH < 6). The samples were incubated with hydrazide beads for one hour at room temperature in the dark with gentle shaking. Beads were then washed to remove any nonspecific binding. PNGaseF (New England Biolabs, Ipswich, MA, USA) was used to detach glycosite-containing peptides from glycans conjugated on the beads. The supernatant containing N-linked glycopeptides were collected and dried using vacufuge. These enriched N-linked glycopeptides were directly used for the mass spectrometric analysis.

### LC-MS/MS analysis

The peptides and enriched glycopeptides were analyzed on Q Exactive HF-X Hybrid Quadrupole-Orbitrap mass spectrometer (Thermo Scientific, Bremen, Germany) interfaced with Dionex Ultimate 3000 nanoflow liquid chromatography system. Peptides were separated on an analytical column (75 μm × 50 cm, RSLC C_18_) at a flow rate of 300 nl/min using a gradient of 8%–35% solvent B (0.1% formic acid in 90% acetonitrile) for 105 min. The total run time was set to 120 min. The mass spectrometer was operated in a data-dependent acquisition mode. The precursor scan MS scan (from m/z 350–1600) was acquired in the Orbitrap at a resolution of 120,000 at 200 m/z. The automatic gain control (AGC) target for MS1 was set as 3 × 10^6^ and ion filling time set at 50 ms. The most intense ions with charge state ≥ 2 was isolated and fragmented using HCD fragmentation with 32% normalized collision energy and detected at a mass resolution of 45,000 at 200 m/z. The AGC target for MS/MS was set as 5 × 10^5^ and ion filling time set at 150 ms, while dynamic exclusion was set for 30 s with a 10-ppm mass window.

### Data analysis

The acquired mass spectrometry data were searched against Human RefSeq protein database (version 81, containing protein entries with common contaminants) using SEQUEST search algorithm through Proteome Discoverer platform (version 2.1, Thermo Scientific). Following search parameters were used: two missed cleavages allowed, trypsin as cleavage enzyme, a tolerance of 10 ppm on precursors and 0.02 Daltons on the fragment ions. Fixed modification includes: carbamidomethylation at cysteine, TMT 10-plex (+229.163) modification at N-terminus of peptide and lysine and variable modification as oxidation of methionine and deamination of asparagine and glutamine. Data was also searched against a decoy database and filtered with a 1% false discovery rate (FDR). Glycopeptides were further filtered for consensus sequence of N-linked glycosylation motif NXS/T. Protein centric glycosylation site were inferred using in house PERL script. The relative abundance of N-glycoproteins was calculated. The mass spectrometry data generated from this study have been deposited to the ProteomeXchange Consortium (http://www.proteomexchange.org) *via* PRIDE partner repository with the dataset identifier PXD020078.

### Statistical analysis

For the identification of glycopeptides differentially glycosylated between NMIBC and MIBC, two sample “*t*-test” with unequal variances (*p* < 0.05) was used. This led to the identification of a list of glycoproteins which were further used for pathway and network analysis.

### Reactome pathway analysis

Reactome analysis tool (http://www.reactome.org) was used to identify the enriched pathways in the NMIBC and MIBC patients using the set of differentially glycosylated peptides (*p* ≤ 0.05) (https://www.reactome.org/PathwayBrowser/#TOOL=AT).

## SUPPLEMENTARY MATERIALS




