# Supplementary material for: Urinary glycoproteomic profiling of non-muscle invasive and muscle invasive bladder carcinoma patients reveals distinct N-glycosylation pattern of CD44, MGAM, and GINM1
Source: Oncotarget. 2020 Aug 25;11(34):3244–55. doi: 10.18632/oncotarget.27696 (PMC7456616; doi:10.18632/oncotarget.27696)
Supplement: Supplementary file 1 [file oncotarget-11-3244-s001.pdf]

Urinary glycoproteomic profiling of non-muscle invasive and muscle invasive bladder carcinoma patients reveals distinct N-glycosylation pattern of CD44, MGAM, and GINM1

SUPPLEMENTARY MATERIALS

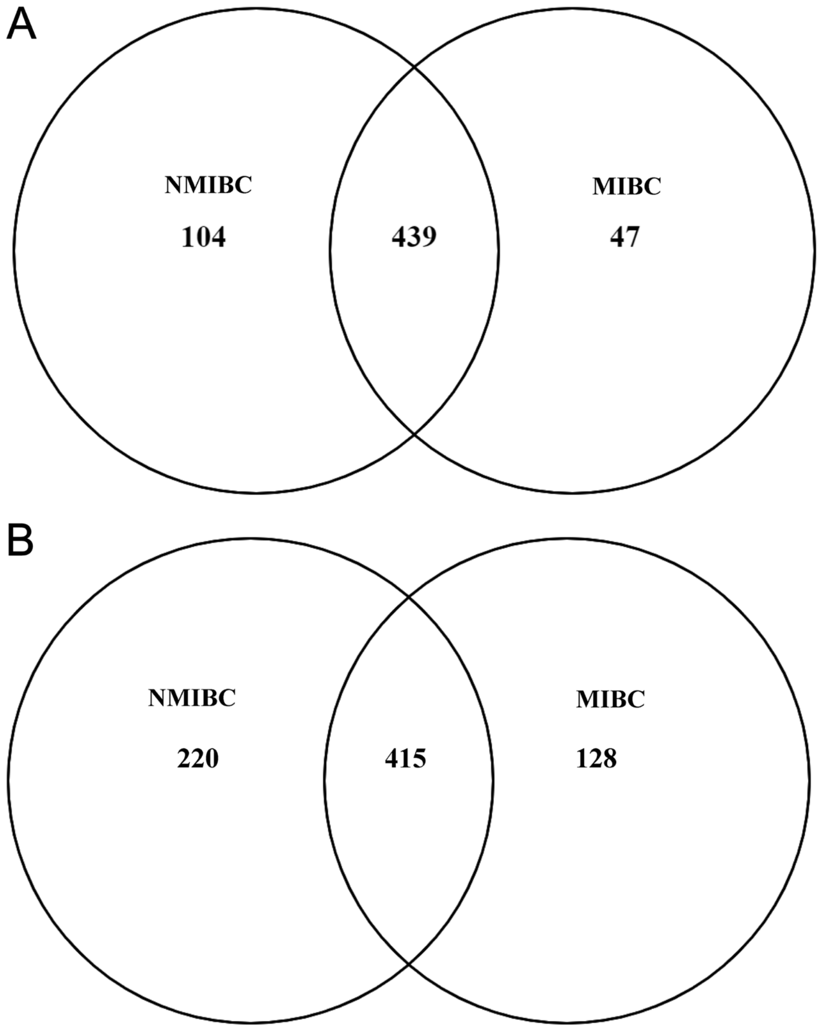

Supplementary Figure 1: (**A**) Mass spectrometric analysis of NMIBC (*n* = 5) and MIBC (*n* = 4) samples resulted in identification of 590 proteins. (**B**) SPEG based N-glycoproteomic analysis of NMIBC (*n* = 5) and MIBC (*n* = 4) samples resulted in identification of 763 N-glycopeptides.

Supplementary Table 1: Overview of all the bladder cancer patients from which urine was obtained

Patient Id	Age	Gender	Clinicopathological staging
T1_31	56	Male	T1
T1_37	83	Male	T1
T1_38	63	Male	T1
T1_47	64	Male	T1
T1_50	64	Male	T1
T2_40	65	Male	T2
T2_46	38	Female	T2
T2_57	38	Male	T2
T2_74	55	Male	T2

Supplementary Table 2: Quantification of N-glycopreotmics of NMIBC patients and healthy cohort. See Supplementary Table 2

Supplementary Table 3: Total proteomics data for Non-muscle invasive bladder carcinoma patients and healthy cohorts. See Supplementary Table 3

Supplementary Table 4: Quantification of N-glycoproteomics of MIBC patients and healthy cohort. See Supplementary Table 4

Supplementary Table 5: Total proteomics data for-muscle invasive bladder carcinoma patients and healthy cohorts. See Supplementary Table 5

Supplementary Table 6: Commonly glycosylated peptides between NMIBC and MIBC with their fold change values**.** See Supplementary Table 6

Supplementary Table 7: Top 5 pathways enriched in NMIBC and MIBC patients. See Supplementary Table 7
